# Supplementary material for: Improvement of Gene Delivery and Mutation Efficiency in the CRISPR-Cas9 Wheat (Triticum aestivum L.) Genomics System via Biolistics
Source: Genes (Basel). 2022 Jun 30;13(7):1180. doi: 10.3390/genes13071180 (PMC9318839; doi:10.3390/genes13071180)
Supplement: Supplementary file 1 [file genes-13-01180-s001.zip › genes-1748108-supplementary.pdf]

Table S1. Primer sets used for selection of transgenic plants and for mutation in genome-edited plants

Name	Sequence	Band Size (bp)
HPT 9F	TCG AAA AGT TCG ACA GCG TCT	815
HPT 10R	AGC TGC ATC ATC GAA ATT GCC	
dsRED 1F	AAC GGC CAC GAG TTC GAG ATC	571
dsRED 2R	GCT CCA CGA TGG TGT AGT CC	
OsUbi 2F	CTC GAA ATT TCC CAA CCG CTC G	1441
mCas9 1R	ACA GAG TTG GTG CCG ATG TCC A	
PDS A1F	ATG GTT AGT TGA TTT AAA GGG	687
PDS A4R	CTT TGT CCT AAG CTA TTA CGA	
PDS B2F	AAT GTT TGG TTG TAA GTT AAG	666
PDS B5R	TAT TAC GGT TAT TAT CAT CAG	
PDS D3F	AAT GTT TGG TTC TAA GTT AAC	676
PDS D6R	CTT TGT CCT AAG CTA TTA CGG	

Table S2. Microcarrier size effect on DsRED co-expression efficiency

Gold Particle Size	# IEs Bombarded	# Transgenic Events	# DsRED-expressing Events	Co-expression Efficiency
0.4 μm	328	35	5	14.3%
0.6 μm	327	74	24	32.4%
1.0 μm	354	32	15	46.9%

*Co-expression efficiency = (# DsRED-expressing events/# transgenic events) x 100%
